# Mechanistic insights into carbon steel corrosion inhibition by benzodiazepine derivatives in hydrochloric acid

**DOI:** 10.1039/d6ra00049e

**Published:** 2026-04-21

**Authors:** N. Timoudan, L. Chahir, A. Marzaq, R. Saddik, S. Tighadouini, I. Warad, B. Dikici, Ahmed A. Farag, F. Bentiss, A. Zarrouk

**Affiliations:** a Laboratory of Molecular Spectroscopy Modelling, Materials, Nanomaterials, Water and Environment, CERNE2D, Faculty of Sciences, Mohammed V University Av. Ibn Battuta. P. O. Box 1014 Rabat Morocco nadia.timoudan1995@gmail.com a.zarrouk@um5r.ac.ma azarrouk@gmail.com +212673923069 +212665201397; b Laboratory of Organic Chemistry, Materials, Electrochemistry and Environment, Faculty of Sciences Ain Chock, Hassan II University BP 5366 Casablanca Morocco; c Department of Chemistry, AN-Najah National University P. O. Box 7 Nablus Palestine; d Ataturk University, Department of Mechanical Engineering 25240 Erzurum Turkey; e Egyptian Petroleum Research Institute (EPRI) Cairo 11727 Egypt; f Laboratory of Catalysis and Corrosion of Materials, Faculty of Sciences, Chouaib Doukkali University P. O. Box 20 M-24000 El Jadida Morocco

## Abstract

The protection of carbon steel from aggressive acidic environments remains a critical challenge in industrial and defence-related applications. In this study, two newly synthesised benzodiazepine derivatives, namely 9-ethyl-2,3,4,9-tetrahydrobenzo[*b*]cyclopenta[*e*][1,4]diazepin-10(1*H*)-one (SR_3_) and 1-ethyl-4-phenyl-1*H*-benzo[*b*][1,4]diazepin-2(3*H*)-one (SR_4_), were investigated as corrosion inhibitors for carbon steel in 1 M HCl solution. Electrochemical measurements, including potentiodynamic polarisation and electrochemical impedance spectroscopy, were complemented by comprehensive surface and spectroscopic analyses (SEM, EDS, AFM, XRD, contact angle, and UV-Vis). Both compounds exhibited high inhibition efficiencies, reaching 85.1% for SR_3_ and 92.8% for SR_4_, with adsorption behaviour following the Langmuir isotherm model. Polarisation results indicated a mixed-type inhibition mechanism, while surface analyses confirmed the formation of a compact and adherent protective film on the steel surface. Density functional theory and molecular dynamics simulations provided molecular-level insights into the adsorption configurations and interaction strength of the inhibitors on the Fe(110) surface, in good agreement with the experimental observations. Overall, these findings demonstrate the effectiveness of benzodiazepine-based frameworks as promising molecular inhibitors for carbon steel corrosion in acidic media.

## Abstract 2

SR_3_ and SR_4_ benzodiazepine derivatives effectively inhibit carbon steel corrosion in 1 M HCl. Surface analyses confirm adsorption of the inhibitors on the carbon steel surface. DFT and MD simulations elucidate the inhibition mechanism.

## Graphical abstract

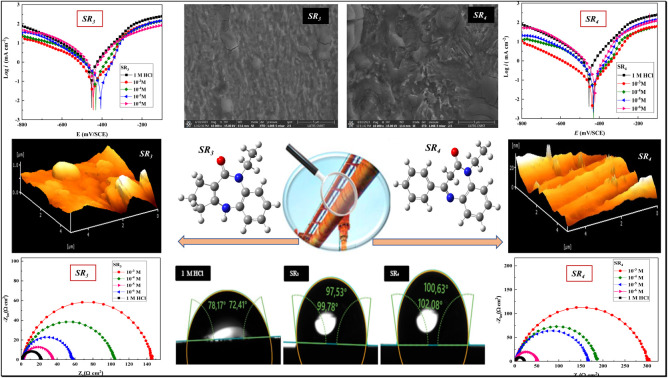

## 1. Introduction

Every year, corrosion of metals and alloys causes major economic losses, especially in industry. Owing to its mechanical characteristics, carbon steel is frequently employed as a base material for various types of construction. However, corrosion problems can unexpectedly affect the profitability of these projects, as the decision to use this material is based on the initial cost. Given its importance, researchers, engineers, theoreticians, and electrochemists are analyzing its resilience to corrosion in harsh conditions.

On the other hand, many chemical processes and industrial procedures, including pickling, cleaning, localised-deposit removal, and acidification of oil wells, rely heavily on acid solutions, particularly hydrochloric acid. Today, corrosion prevention is both a scientific & technical research field and a financial requirement. The search for new, environmentally friendly inhibitors is justified by banning harmful modifiers such as chromates and nitrites in anti-corrosion treatments.

The presence of heteroatoms (N, O, S, and P), aromatic rings, functional/groups, a large number of π-electron-containing bonds, and electron density are all crucial factors that affect how efficiently these organic molecules inhibit.^1–4^ Amine derivatives are especially valued because they can create very effective chemicals as corrosion inhibitors and add new functional groups to their structures. Both physical and chemical adsorption depend on the molecules' electrostatic interaction with the metal surface and the d-orbital charge sharing between the heteroatoms and aromatic ring of organic compounds, which makes it easier for the molecules to adhere to the metal surface.

Recent studies on stainless steel have highlighted the importance of alloy composition and surface interactions in corrosion resistance.^5,6^ While these studies focus on carbon steel, the insights into microstructural stability and surface interactions are also relevant for understanding corrosion mechanisms in carbon steel. Such knowledge supports the evaluation of organic inhibitors, including benzodiazepine derivatives, by emphasizing the importance of adsorption and protective film formation in acidic media.

It seems that benzodiazepine molecules are the most effective at preventing corrosion. In fact, a benzodiazepine, which is sometimes shortened to “BZD” or simply “benzo,” is a psychotropic medication whose chemical structure is based on the combination of a diazepine and a benzene ring. Leo Sternbach discovered the first benzodiazepine, chlordiazepoxide (Librium), in 1955. Hoffmann-La Roche put it on the market in 1960 and introduced diazepam (Valium) in 1963.^7^ Furthermore, there is a dearth of information regarding the application of benzodiazepine derivatives in acidic settings.^8–11^

This study evaluates the ability of two synthetic benzodiazepine compounds, specifically 9-ethyl-2,3,4,9-tetrahydrobenzo[*b*]cyclopenta[*e*][1,4]diazepin-10(1*H*)-one (SR_3_) & 1-ethyl-4-phenyl-1*H*-benzo[*b*][1,4]diazepin-2(3*H*)-one (SR_4_), to prevent corrosion of C.steel in a solution of 1 M HCl. Electrochemical impedance spectroscopy, contact angle, SEM/EDS, AFM, XRD, isothermal computations, potentiodynamic polarization curves, and UV-visible examination were among the methods used at different phases to evaluate the efficacy of these substances. DFT computations and molecular-dynamics/simulations (MD) were used to ascertain the connection between the molecular structures of the studied derivatives and their capacity to inhibit corrosion. Along with determining the adsorption & thermodynamic-parameters, the adsorption mechanism was also suggested and discussed. The molecular structures of the SR_3_ & SR_4_ compounds examined are shown in Table 1.

**Table 1:** Nomenclatures, chemical structures, & abbreviations of benzodiazepine derivatives

Nomenclature	Molecular/structure	Abbreviation
9-Ethyl-2,3,4,9-tetrahydrobenzo[*b*]cyclopenta[*e*][1,4]diazepin-10(1*H*)-one	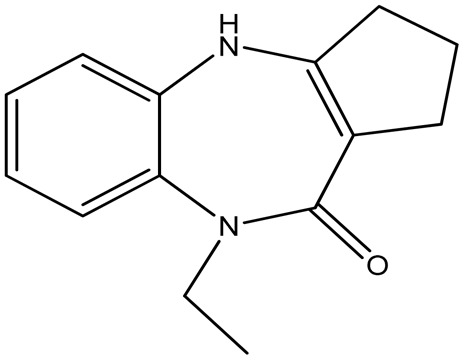	SR_3_
1-Ethyl-4-phenyl-1*H*-benzo[*b*][1,4]diazepin-2(3*H*)-one	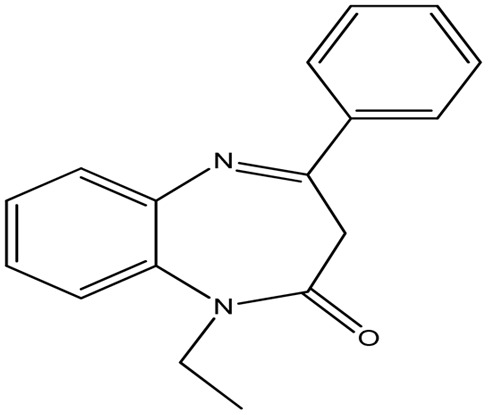	SR_4_

## 2. Experimental

### 2.1. Materials

Corrosion tests were conducted using C.stl plates that had the composition below (in weight percentage): S-0.01600%, Cr-0.07700%, Ti-0.011000%, Ni-0.059000%, Co-0.009000%, Cu-0.16000%, C-0.37000%, Si-0.230000%, Mn-0.680000%, and iron as the predominant element. Acetone was used to clean the C.stl plates for electrochemical investigations after they were mechanically abraded using SiC sandpaper grades 180–1200. The plates were then allowed to dry at room temperature. The test solution was diluted an analytical-grade 37% HCl solution with double-distilled water to create a 1 M HCl environment.

### 2.2. Electrochemical assessments

A typical three-electrode cell is formed by immersing three electrodes in the electrolyte in all electrochemical test experiments. This arrangement consists of a C.steel working electrode (WE) with a surface area of 1 cm^2^ exposed to the electrolyte, a saturated calomel electrode (SCE) as the reference electrode, and a platinum electrode (Pt) as the counter-electrode. The computer-connected PGZ100 potentiostat was used for the tests, and the ‘VoltaMaster 4.0’ electrochemical analysis program was used to gather electrochemical data. The C.stl's open circuit potential (OCP) was stabilized after 30 minutes of immersion in hydrochloric acid 1 M. The potentiodynamic-polarization analysis was set between −800 and −100 mV per SCE.

Electrochemical-impedance tests were conducted in the 100 kHz to 10.0 mHz range with a signal amplitude of 10 mV at a 0.5 mV s^−1^ scan rate. Volta-master transforms the data from electrochemical studies into semicircular, which are fitted using ZView software.

### 2.3. Surface investigations

• Using JEOL JSM-IT 100 equipment, SEM was employed to characterize the C.steel surface before and after a 24 hours immersion in a 1 M HCl solution, with or without adding 1 × 10^−3^ M benzodiazepines. Since our team has already published a study with comparable conditions, we have used the blank's results in both the presence and absence of HCl without inhibitor.^12^

• Atomic Force Microscopy (AFM) by a Hitachi 5100N was used to observe the deposited film shape at *T* = 303 K. Using an imaging methods system, the AFM experiments are conducted by immersing the electrode in 1 M HCl for twenty-four hours, in the presence and absence of 1 × 10^−3^ M of the two compounds being studied.

• Contact angle analysis was performed using the Biolin Scientific Attension Theta device. In contrast, an X-ray diffraction (XRD) assessment was conducted using a Shimadzu 6100 diffractometer (JEOL/Model JSM-IT) operating at a 20 kV voltage.

The electrochemical and surface examination findings for the non-inhibited solution used in this study are identical to those in our previous publication. This is significant because the same equipment and setup were used for the trials.^13^

### 2.4. UV-visible analysis

The effectiveness of corrosion inhibitors SR_3_ and SR_4_ for C.stl has been investigated using UV-visible spectroscopy, a technique based on light absorption characteristics at particular wavelengths. To analyze the interactions between these organic molecules & the surface/C.steel in a 1 M HCl medium, absorption tests of the solution were conducted both with and without a specimen of C.stl at a concentration of 1 × 10^−3^ M for SR_3_ and SR_4_ molecules. The lengths of the onde were measured using a UV-visible spectrophotometer (JASCO series V-730) that covered the 190–600 nm range.

### 2.5. DFT computations

Gaussian 09 software was used to perform theoretical calculations in both gas phase and solution, utilizing density-functional/theory (DFT) with the hybrid function B3LYP,^14–16^ Water's effect as a solvent was considered using the polarizable continuum model (PCM), as electrochemical corrosion usually takes place in an aquatic environment. For optimization, the bases 6-31G(d,p) & 6-311+G(d,p) were employed for all species examined.

This article presents quantum chemical studies within the framework of Kohn–Sham density-functional/theory. The equations of this theory have been employed to estimate various global quantum chemical descriptors, such as ionization potential (*I*), electronic affinity (*A*), energy gap (Δ*E*), electronegativity (*χ*), hardness (*η*), and softness (*σ*), among others.^17–20^

The following equations are used to calculate the overall energy change of back-donation (Δ*E*_b–d_) and the number (fraction) of electrons transferred (Δ*N*) from the inhibitor the inhibitor to the metal/surface.1
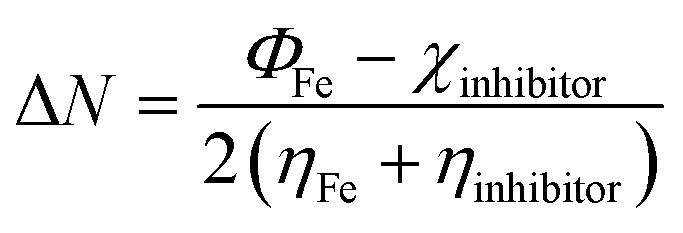
2
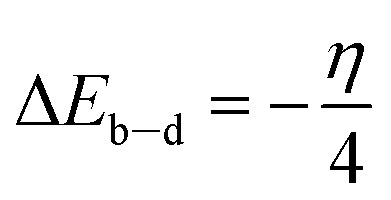
where *Φ*_Fe_ & *η*_Fe_ which have values of 4.820 eV mol^−1^ & 0.00 eV mol^−1^, respectively, represent the metal's work function and absolute hardness.

### 2.6. MD computational studies

The Forcite module, which was integrated into the Materials Studio 8.0 program created by BIOVIA Inc., carried out molecular dynamics simulation.^21,22^ A 40-high vacuum slab and periodic boundary conditions were used to model the interaction between the Fe (110) surface and the macromolecular matrix of benzodiazepine derivatives in a box measuring 32.27 × 32.27 × 30.13 Å^3^. The simulated species, such as 500H_2_O, 5Cl^−^, 5H_3_O^+^, and benzodiazepine-derivatives (SR_3_, SR_4_), interact with the Fe (110) surface *via* the COMPASS force environment. Using the canonical ensemble (*NVT*) with a step count of 1 fs and a simulation length of 1000 ps, the Andersen thermostat controls the temperature at 303 K during this simulation.^23^

## 3. Results & discussion

### 3.1. Polarization curves

**Fig. 1:**
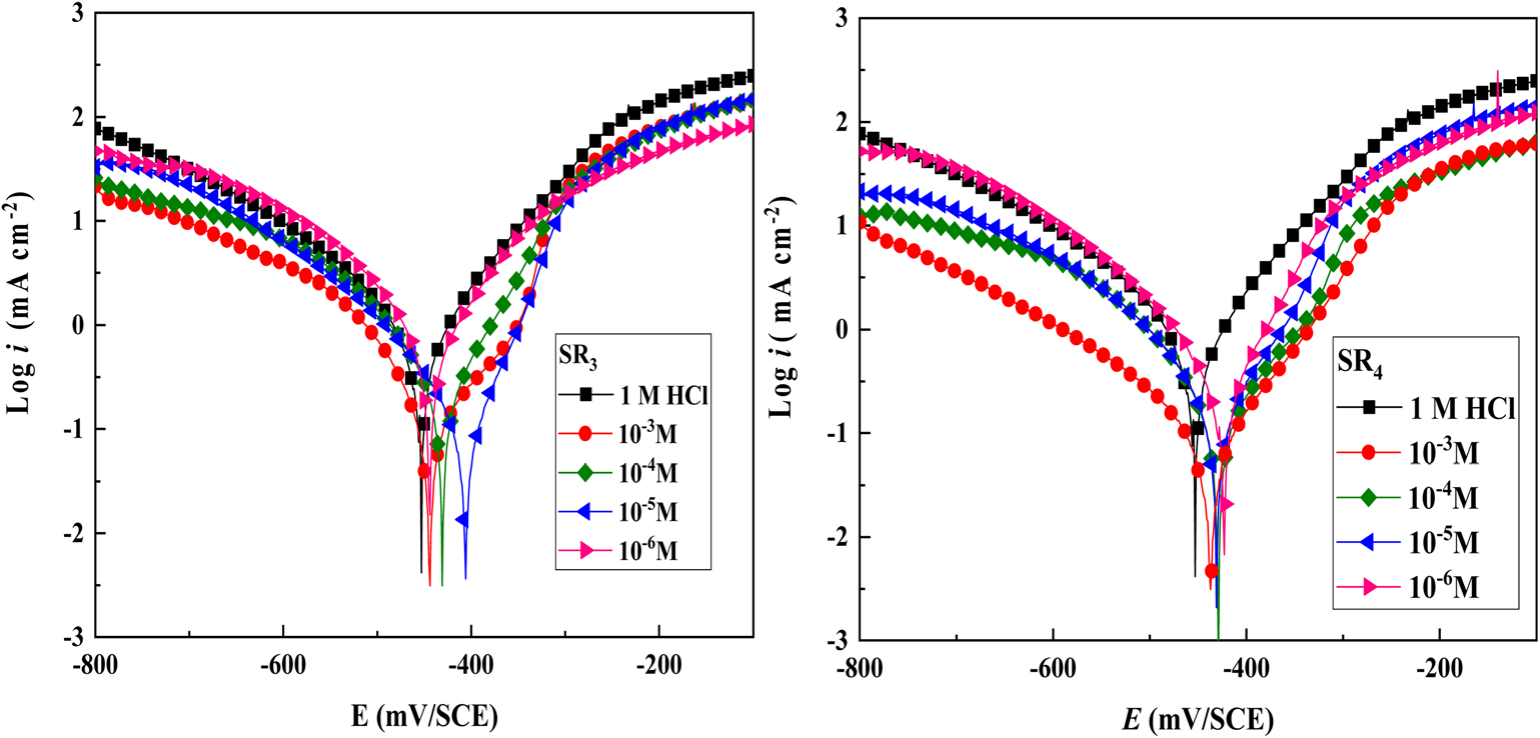
Polarisation plots of C.stl in hydrochloric acid at various concentrations at 303 K.

Fig. 1 shows the polarization curve of C.steel that was submerged in a corrosion solution for half an hour at 303 K. Both the cathodic & anodic branches are reduced using SR_3_ and SR_4_. The benzodiazepine derivatives appear to function as mixed-type inhibitors, as evidenced by the maximum measured shift of 51.5 mV, which is below the threshold of 85 mV.^24^ All PDP curves showed parallel cathodic branches, suggesting that the cathodic reaction mechanism was unaffected by the existence of benzodiazepine derivatives.^25^ Furthermore, Fig. 1 shows that incorporating benzodiazepine compounds into 1 M HCl blocked both the anodic & cathodic branches. As the concentration rose, the influence of these derivatives on current densities became more noticeable. C.steel dissolving and hydrogen reduction at the corresponding reaction sites is significantly reduced due to the suppression of both anodic & cathodic/reactions on the C.steel/surface.^26^

According to the extrapolated/electrochemical values presented in Table 2, the incorporation of the benzodiazepine derivatives notably decreases *i*_corr_ when compared to the 1 M hydrochloric acid environment. At 10^−3^ M, the inhibition efficiency (*η*%) rises with the derivatives' concentration, peaking at 87.9% for SR_3_ and 94.2% for SR_4_. According to the extrapolated electrochemical values presented in Table 2, the incorporation of benzodiazepine derivatives significantly decreases *i*_corr_ compared to the 1 M HCl environment. At 10^−3^ M, the inhibition efficiency (*η*%) increases with the derivatives' concentration, reaching 87.9% for SR_3_ and 94.2% for SR_4_. While the cathodic branches of the polarization curves appear roughly parallel, the observed changes in the cathodic Tafel slopes (*β*_c_, Table 2) indicate that the cathodic reaction or the rate-determining step may be affected by the presence of the inhibitors. The maximum measured shift corresponds to the offset of the corrosion potential (Δ*E*_corr_), which remains below the 85 mV threshold, confirming that the inhibitors act as mixed-type, with a significant influence on the hydrogen evolution process.

**Table 2:** Polarization values for C.stl in 1 M HCl, in the non-existence and existence of the addition of benzodiazepine derivatives

Environment	Conc.	−*E*_cor_ (SCE) (mV)	*i*_corr_ (µA cm^−2^)	*β*_a_ (mV dec^−1^)	−*β*_c_ (mV dec^−1^)	*η*_PPD_ (%)
HCl	1 M	456.3	1104.1	112.8	155.4	—
SR_3_	10^−3^	443.1	133.3	68.5	79.7	87.9
	10^−4^	429.8	216.3	70.7	89.8	80.4
	10^−5^	405.2	428.1	75.5	165.1	61.2
	10^−6^	442.7	723.7	95.2	109.3	34.4
SR_4_	10^−3^	435.6	63.5	56.1	105.1	94.2
	10^−4^	427.4	118.5	68.8	76.1	89.2
	10^−5^	429.1	136.1	84.9	82.5	87.6
	10^−6^	422.2	176.8	88.5	69.9	83.9

The addition of benzodiazepine derivatives also leads to a decrease in the anodic Tafel slopes (*β*_a_), suggesting an effect on the anodic dissolution reaction.^27^ This behavior implies that benzodiazepine molecules adsorb onto the C.stl/surface, creating an insulating layer & lowering the number of available active sites. Moreover, the heteroatoms of the benzodiazepine molecules (oxygen and nitrogen) may encourage the development of a shield that blocks the dissolution of C.stl. Consequently, when fewer active sites occur where the corrosion process occurs, the rate of anodic dissolution and hydrogen release is slowed.^28,29^ The detailed adsorption behavior and the formation of a protective film on the steel surface are discussed in the following section, based on Langmuir isotherm analysis and surface characterization techniques.

### 3.2. EIS investigation

**Fig. 2:**
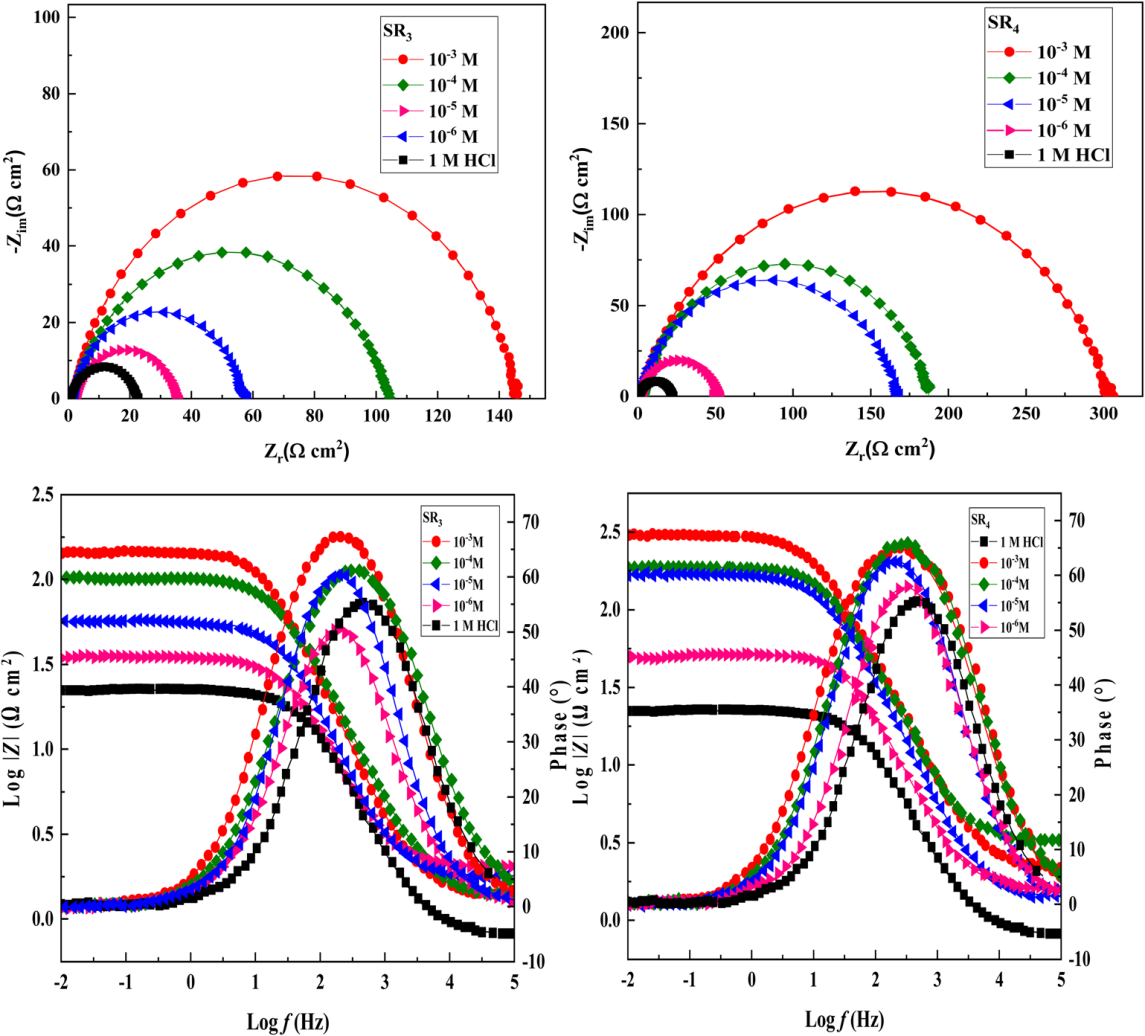
Nyquist and Bode diagrams for C.stl in uninhibited and inhibited conditions.

Fig. 2 displays the Nyquist & Bode graphs of the EIS curves for C.stl in a hydrochloric acid environment. A charge transfer mechanism is shown by the spectrum's capacitive loop when no additives are present.^30,31^ Furthermore, as the concentration of SR_3_ and SR_4_ rises, the size of the semicircles in the inhibited electrolytes increases significantly. This enlargement indicates the formation of a protective film on the steel surface due to the adsorption of these derivatives. In addition, the phase diagrams in Fig. 2 show a single peak, suggesting that the SR_3_ and SR_4_ corrosion processes occur in a single step, controlled by charge transfer.^32^

**Fig. 3:**
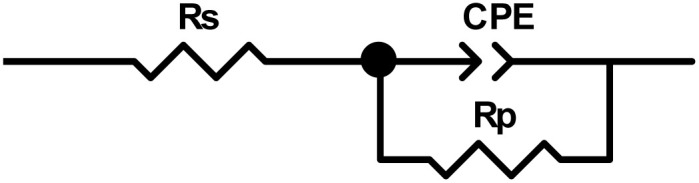
The equivalent circuit.

Modeling the EIS data using the equivalent circuit model allowed for a more thorough examination of the effects of SR_3_ and SR_4_ on C.stl, as seen in Fig. 3. *R*_p_ (polarization-resistance), a constant-phase-element (CPE) that represents the double-layer/capacitance (*C*_dl_) on a heterogeneous/surface, & *R*_s_ (solution-resistance) are among the electrochemical parameters that were determined. When SR_3_ or SR_4_ is present or absent, eqn (3) (ref. 33) describes the impedance.

The matching electrochemical values are shown in Table 3. The findings show that the presence of benzodiazepine derivatives lowers double-layer/capacitance (*C*_dl_) by increasing polarization-resistance (*R*_p_). The widening of the semicircle in the Nyquist diagram, which indicates an elevation of *R*_p_ values in inhibited systems, verifies that SR_3_ and SR_4_ molecules prevent corrosion. The drop in *C*_dl_ parameters, which are usually associated with the thickness of the double-layer, could be due to the adsorption of benzodiazepine derivatives on the C.stl/acid interface. These derivatives have a lower dielectric constant than adsorbed water, which protects against metal corrosion.^34,35^ When compared to the hydrochloric acid environment, Table 3 shows a significant decrease in *R*_p_ with the addition of the benzodiazepine derivatives, while the inhibition efficiency (*η*_SIE_%) improves as the derivative concentration increases. At 10^−3^ M, SR_4_ showed the highest corrosion inhibition (*η*_SIE_% = 92.8%), whereas SR_3_ showed the highest corrosion inhibition (*η*_SIE_% = 85.1%), indicating that benzodiazepine derivatives act as excellent corrosion inhibitors for steel in acidic environments.

**Table 3:** Impedance data for C.stl in hydrochloric acid, both including and excluding various concentrations of benzodiazepine derivatives at 303 K

Environment	Conc. (M)	*R*_s_ (Ω cm^2^)	*R*_p_ (Ω cm^2^)	*Q* (µF s^*n*−1^ cm^−2^)	*n*	*C*_dl_ (µF cm^−2^)	*χ* ^2^	*η*_SIE_ (%)
HCl	1 M	0.83	21.57	293.9	0.845	116.2	0.002	—
SR_3_	10^−3^	1.46	144.4	135.2	0.868	74.3	0.006	85.1
	10^−4^	1.55	102.6	155.8	0.857	78.1	0.008	79.0
	10^−5^	1.70	54.9	182.1	0.851	81.3	0.007	60.8
	10^−6^	1.93	33.1	203.5	0.847	82.5	0.008	35.0
SR_4_	10^−3^	2.17	300.7	67.01	0.875	38.4	0.008	92.8
	10^−4^	3.47	184.2	80.4	0.866	41.9	0.007	88.3
	10^−5^	1.46	166.0	101.5	0.858	51.6	0.008	87.0
	10^−6^	1.10	146.3	123.8	0.856	63.0	0.007	85.3

An excellent method for evaluating a solid surface's inhibitory behavior is to use adsorption isotherms. Based on formula (3), the Langmuir-isotherm with a linear regression coefficient (*R*^2^) and a slope of around one was found to be the best model for predicting the EIS results.3
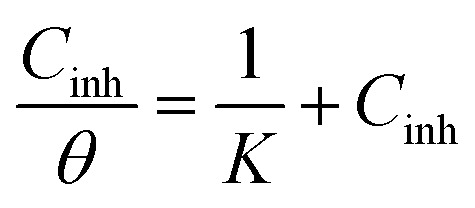
where *K* is the adsorption–equilibrium constant, which is related to the standard-adsorption free energy (Δ*G*_ads_), as given by the following expression:

4 Δ*G*_ads_ = −*RT* ln(55.5 × *K*)

In the equation, water's molarity is 55.5, the temperature is *T*, and the gas constant is *R*.

**Fig. 4:**
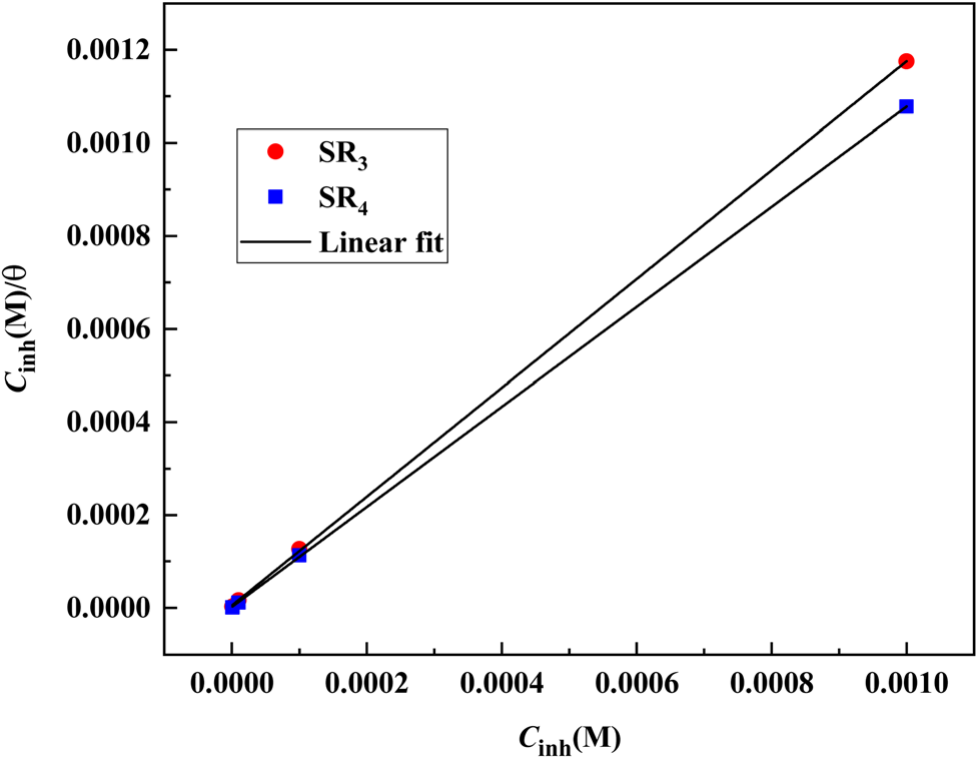
Langmuir-plot for C.stl corrosion in 1 M HCl with benzodiazepine derivatives.

Fig. 4 illustrates the Langmuir isotherm, showing the adsorption of benzodiazepine derivatives onto the surface of carbon steel in a 1 M HCl solution.

The range of Δ*G*_ads_ is −40.7 kJ mol^−1^ to −43.1 kJ mol^−1^, as seen in Table 4. Therefore, when both benzodiazepine equivalents are adsorbed onto the C.stl/surface, chemical adsorption is predominant. Furthermore, the value for SR_4_ is substantially greater and more damaging than SR_3_'s, indicating that SR_4_ provides superior adsorption, in agreement with the higher inhibition efficiency observed in the PDP results. This demonstrates that the mixed-type inhibition behavior observed in the polarization measurements arises from the adsorption of the inhibitor molecules and the formation of a stable protective layer on the steel surface.

**Table 4:** Activation data for C.stl dissolution in hydrochloric acid with and without 10^−3^ M of benzodiazepine derivatives

	*R* ^2^	Slope	*K* (L mol^−1^)	Δ*G*_ads_ (kJ mol^−1^)
SR_3_	0.9999	1.170	1.93 × 10^5^	−40.7
SR_4_	0.9999	1.075	4.879 × 10^5^	−43.1

### 3.3. Effect of temperature

Temperature can cause several changes at the electrolyte/steel interface, including increased surface attack, inhibitor molecule desorption, and even inhibitor breakdown.^36^ In light of this, the development of inhibitor corrosion rates was examined during a 30 minutes immersion in a corrosive solution of hydrochloric-acid at various *T* (303 to 333 K), both with & without the SR_3_ and SR_4_ inhibitors (at a concentration of 10^−3^ M).

**Fig. 5:**
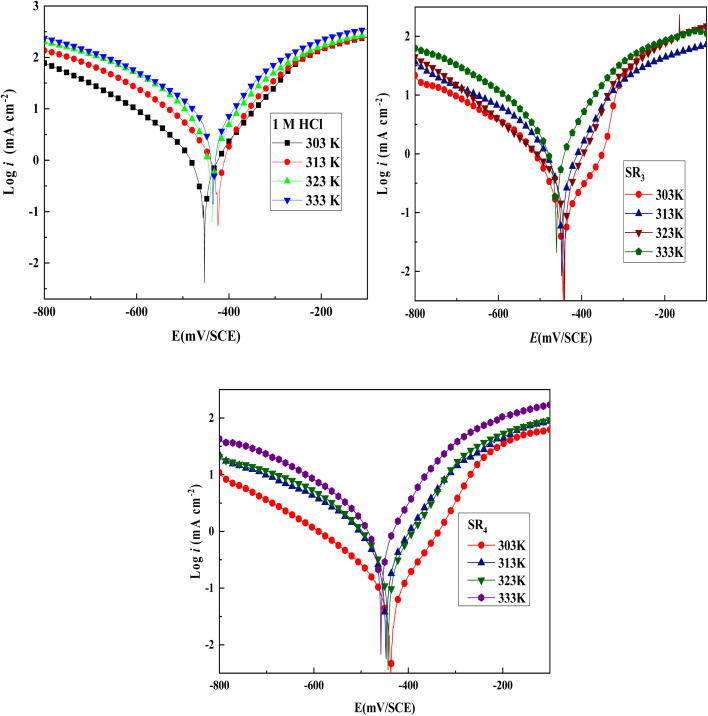
PDP plots in the non-existence and existence of 10^−3^ M SR_3_ & SR_4_ molecules at various temperatures.

The *i*_corr_ current density rises with increasing temperature, as demonstrated by the potentiodynamic polarization curves in Fig. 5. Additionally, Table 5 shows that the percentage inhibition efficiency (IE) for compounds SR_3_ and SR_4_ drops somewhat with temperature, from 87.9% to 75.6% and 94.2% to 84.5%, respectively. This highlights the reduced adsorption of SR_3_ & SR_4_ compounds, especially at elevated temperatures.

**Table 5:** PDP parameters in the non-existence and existence of 10^−3^ M SR_3_ and SR_4_ molecules at different temperatures

Medium	Temp (K)	−*E*_corr_ (SCE) (mV)	*i*_corr_ (µA cm^−2^)	*β*_a_ (mV dec^−1^)	−*β*_c_ (mV dec^−1^)	*η*_pp_ (%)
1 M HCl	303	456.3	1104.1	112.8	155.4	—
	313	423.5	1477.4	91.3	131.3	—
	323	436.3	2254.0	91.4	117.8	—
	333	433.3	3944.9	103.9	134.6	—
SR_3_	303	443.1	133.3	68.5	79.7	87.9
	313	448.2	255.1	66.7	64.6	82.7
	323	441.3	465.1	91.3	165.7	79.3
	333	459.8	962.1	89.6	109.7	75.6
SR_4_	303	435.6	63.5	84.9	105.1	94.2
	313	447.3	154.1	60.5	65.6	89.5
	323	442.1	287.2	87.0	103.6	87.2
	333	458.05	610.6	79.6	104.1	84.5

Although a decrease in inhibition efficiency was observed with increasing temperature, SR_4_ retained a relatively high performance at 10^−3^ M. However, it should be noted that temperature-dependent measurements were carried out only at this concentration. Therefore, broader conclusions regarding adsorption behavior at lower concentrations require further investigation.

An analysis of the literature demonstrates how important temperature is to metal corrosion. Because hydrogen's in acidic environments, the reduction potential typically diminishes, hydrogen depolarization is preferred, and corrosion rates are accelerated. Activation energy (*E*_a_), activation entropy (Δ*S*_a_), and activation enthalpy (Δ*H*_a_) are among the qualities that can be better understood through studies conducted at different temperatures. The Arrhenius equation^37^ often demonstrates a strong correlation between temperature and corrosion rate:5
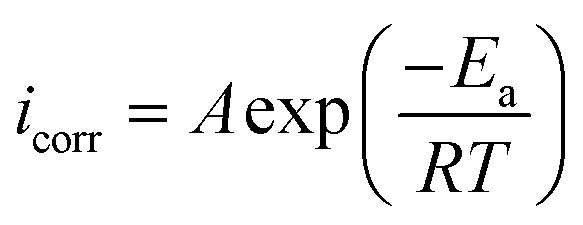
6
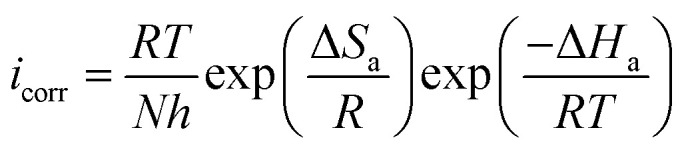


**Fig. 6:**
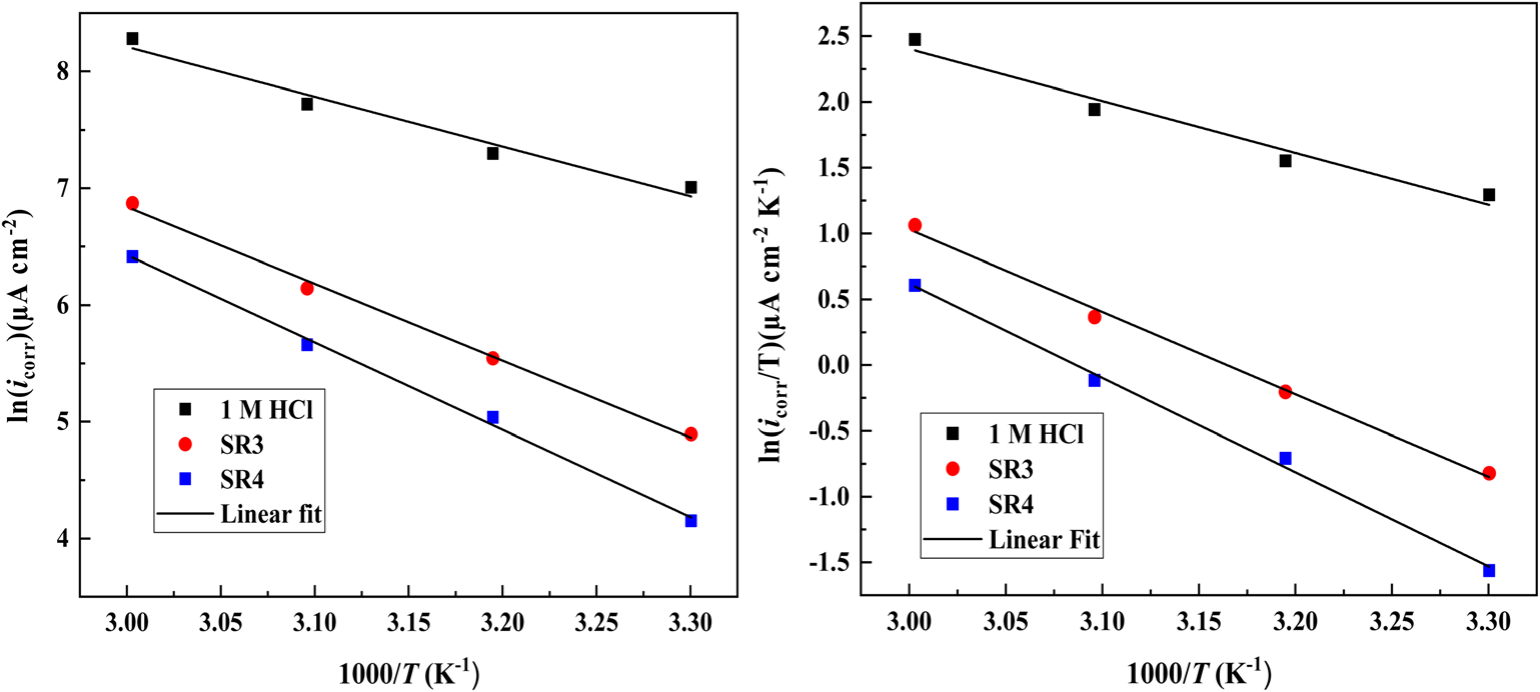
Arrhenius plots for C.stl in uninhibited and inhibited conditions.

In this case, *T* represents the absolute temperature, *R* the gas constant, and *A* the Arrhenius pre-exponential factor. Fig. 6 shows that when ln *i*_corr_ is plotted against 1/*T*, straight lines are produced.

Fig. 6 presents an Arrhenius plot that exhibits a linear relationship with a slope of −*E*_a_/*R*. The calculated activation energy (*E*_a_) for systems incorporating SR_3_ & SR_4_ ({C.stl/SR_3_ or SR_4_/1 M HCl}) is higher compared to the reference medium. An increase in *E*_a_ parameters suggests a reduction in the rate of the corrosion reaction.^38^ In other words, SR_3_ and SR_4_ slow down the breakdown of C.stl in a hydrochloric-acid/environment by decreasing the corrosive-reactivity agents or preventing them from reaching the metal/surface. The positive Δ*H*_a_ values in Table 6 suggest that the dissolution process in C.stl is endothermic. The increase in Δ*S*_a_ when switching from the electrolyte solution to the electrolyte solution in the presence of both inhibitors implies that an increase in disorder occurs when the reactants switch to the activated complex.^39^

**Table 6:** Activation energies for C.stl samples in 10^−3^ M hydrochloric acid environment incorporating varying concentrations of SR_3_ and SR_4_

Medium	*R* ^2^	*E*_a_ (kJ mol^−1^)	Δ*H*_a_ (kJ mol^−1^)	Δ*S*_a_ (J mol^−1^ K^−1^)
Reference	0.9710	35.40	32.70	−79.21
SR_3_	0.994	54.60	52.0	−32.80
SR_4_	0.995	62.10	59.50	−13.71

### 3.4. SEM/EDS analysis

**Fig. 7:**
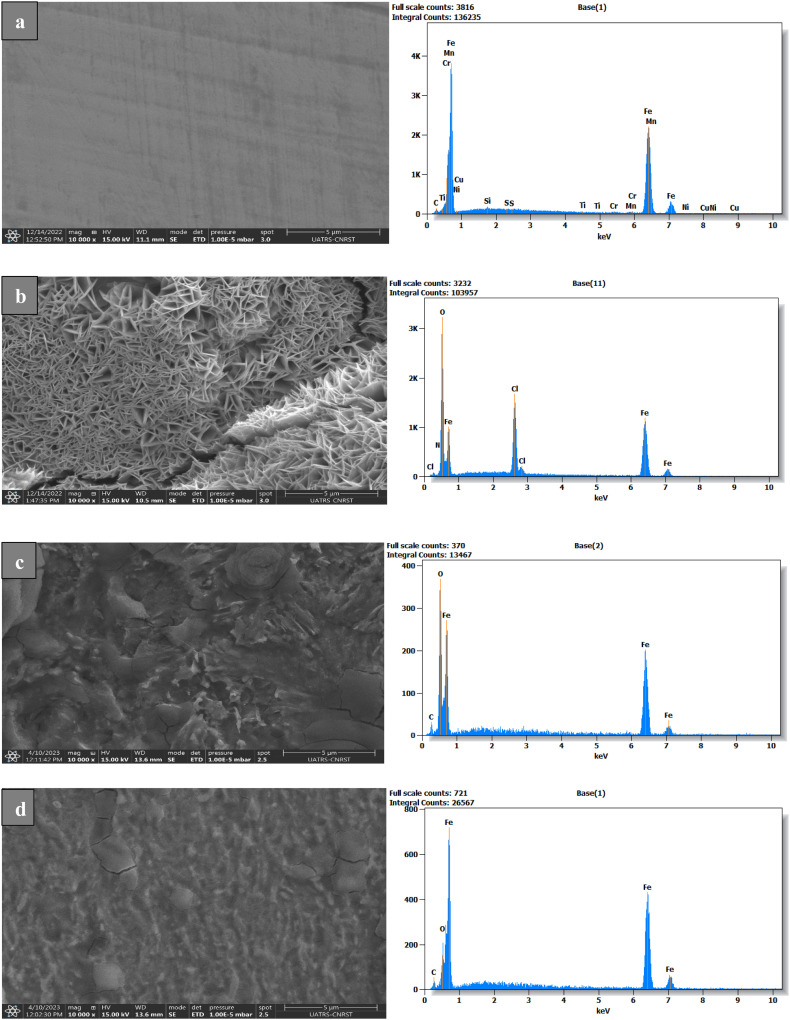
SEM/EDS images of C.stl alone (a), with hydrochloric acid (b), and with 10^−3^ M SR_3_ (c) & SR_4_ (d) after 24 hours of submersion.

The surface topography of samples can be effectively analyzed using the scanning electron microscope (SEM). HCl containing benzodiazepine derivatives at 10^−3^ M or pure HCl was applied to C.stl samples for 24 hours. Uninhibited and inhibited samples' SEM images are displayed in Fig. 7a–c, respectively. Fig. 7a shows C.stl not exposed to HCl, whose surface appears smooth and homogeneous, with only minor imperfections and no obvious signs of corrosion. This is characteristic of a metal not subjected to a corrosive environment. In contrast, Fig. 7b shows the steel after exposure to 1 M HCl without an inhibitor, where the surface is heavily damaged, with deep cavities, pitting, and a rough structure. These alterations are the result of severe corrosion due to the dissolution of iron under the effect of the acid, accompanied by the formation of corrosion products (iron oxides and chlorides). The surface shows a significant loss of material, confirming a rapid and aggressive attack in the absence of an inhibitor. Fig. 7c, showing the steel in the presence of 1 M HCl + 10^−3^ M SR_3_, shows a less damaged surface than in Fig. 7b, although irregularities and porosities are still visible. The SR_3_ inhibitor appears to have limited acid attack, but signs of corrosion remain, suggesting partial protection. A protective film formed by the adsorption of SR_3_ could be present in the form of deposits on the metal surface. Finally, Fig. 7d shows the steel in the presence of 1 M HCl + 10^−3^ M SR_4_, where the surface is much more protected compared to the previous conditions. Very few cavities or pits are visible, indicating that SR_4_ has formed a more effective protective layer, reducing acid attack. The homogeneous structure observed suggests stronger adsorption of SR_4_, which significantly limits metal degradation. These observations confirm that SR_4_ offers better protection than SR_3_ against corrosion of carbon steel in an acidic environment.

In the case of carbon steel alone, not exposed to HCl (Fig. 7a), the dominant signal corresponds to iron (Fe), the main constituent of steel. Faint traces of oxygen (O) are detected, probably due to a thin layer of natural oxidation on the surface. The absence of chlorine (Cl) and other elements indicates that the steel has not undergone any corrosion or adsorption of inhibitors. When exposed to 1 M HCl without an inhibitor (Fig. 7b), the EDS reveals a significant decrease in the Fe signal, reflecting partial dissolution of the metal by the acid. Simultaneously, an increase in the Cl signal is observed, confirming the formation of corrosion products, notably FeCl_3_, by reaction between the iron and the acid. In addition, the increased presence of oxygen (O) indicates significant oxidation, forming iron oxides and hydroxides. These observations confirm severe and unprotected corrosion of the steel in this acidic environment. With the addition of 10^−3^ M SR_3_ in 1 M HCl medium (Fig. 7c), we observed an increase in the Fe signal compared with the condition without inhibitor, indicating a reduction in metal dissolution due to the protective effect of SR_3_. In addition, a decrease in the Cl signal suggests that SR_3_ limited the formation of corrosion products. The appearance of new elements, such as C and N, confirms the adsorption of SR_3_ to the metal surface. However, the residual presence of Cl and O indicates that inhibition is not complete, and that traces of corrosion remain. Finally, in the presence of 10^−3^ M SR_4_ (Fig. 7d), inhibition is even more marked. The Fe signal is more intense than in the case of SR_3_, indicating better protection against dissolution. The Cl signal is strongly reduced, showing that SR_4_ limits FeCl_3_ formation more than SR_3_. In addition, the marked appearance of C and N suggests more efficient adsorption of SR_4_, reinforcing the protective layer formed on the steel surface. The decrease in the O signal also indicates better control of oxidation, reducing the formation of iron oxides.

### 3.5. AFM

**Fig. 8:**
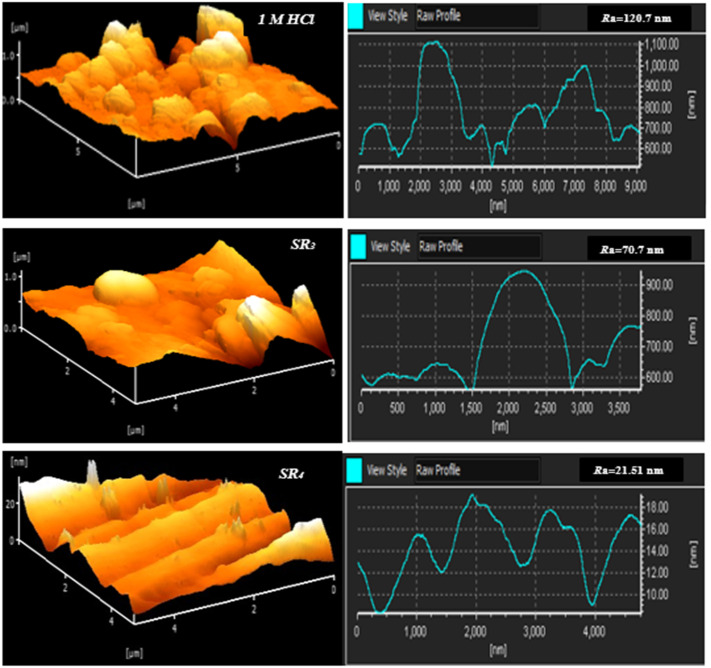
AFM images of C.stl with and without benzodiazepine derivatives.

The AFM images (Fig. 8) reveal that the surface/C.stl becomes highly rough Following contact with hydrochloric acid. In contrast, the existence of benzodiazepine derivatives suggests a protective effect through forming an adsorbed layer. Samples containing these derivatives exhibited reduced surface roughness, with SR_3_ showing an average roughness of 70.7 nm and SR_4_ at 21.51 nm, compared to the untreated steel, which had a roughness (*R*_a_) of 120.7 nm. This roughness reduction highlights the derivatives' effectiveness, with SR_4_ demonstrating superior performance due to better adsorption. These AFM results are consistent with findings from other surface analysis techniques.

### 3.6. Contact angle (CA)

**Fig. 9:**
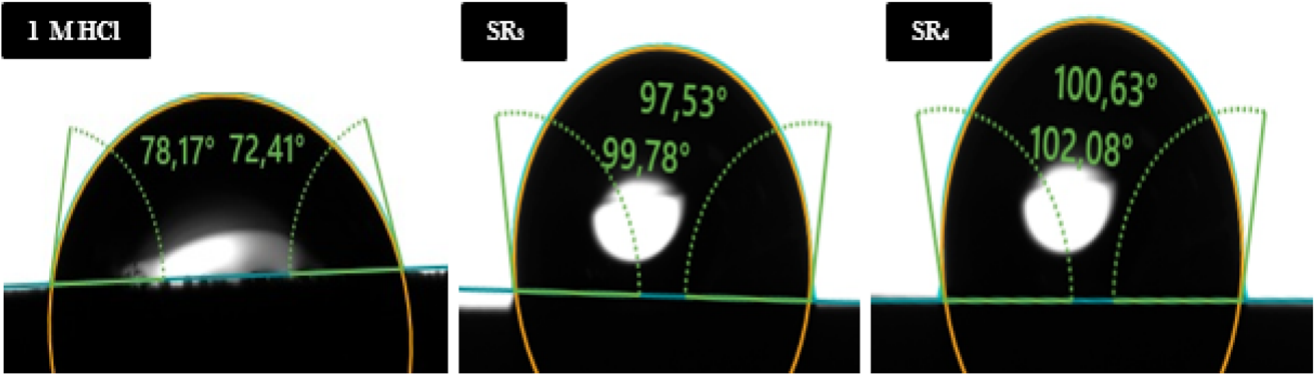
Contact angle measurements of C.steel submerged in hydrochloric acid for 6 hours, both in the non-existence and existence of 1 × 10^−3^ M of SR_3_ & SR_4_.

The results of CA measurement suggests that the angle is higher when SR_3_ & SR_4_ are present than when they are absent (Fig. 9). CA analysis shows a decrease in the metal/surface's contact with water, indicating that these compounds make the surface more hydrophobic by creating a protective organic layer. With SR_4_ outperforming SR_3_, contact angles rise from 78.17° for the untreated surface to 99.78° and 102.08° for SR_3_ & SR_4_, respectively. The efficacy of the protective barrier produced by benzodiazepine derivatives is confirmed by a contact angle larger than 90°, which denotes a hydrophobic surface.

### 3.7. XRD examination

**Fig. 10:**
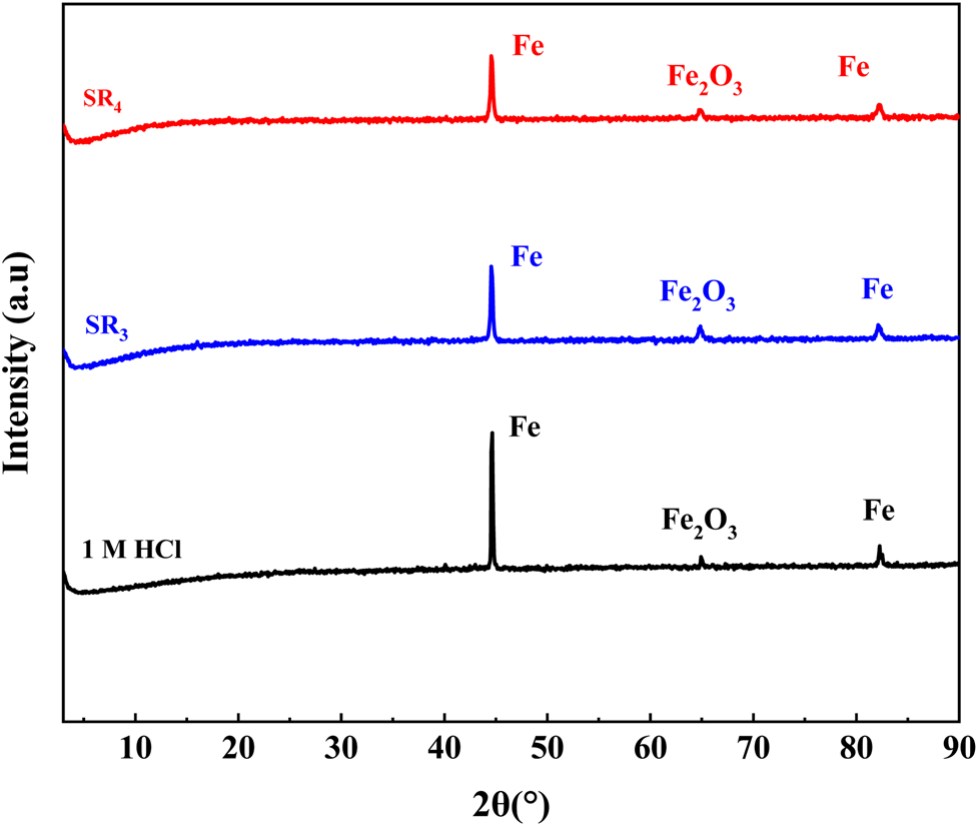
XRD-patterns of corroded and inhibitor-treated C.stl substrates.

Diffractograms of C.stl (Fig. 10) show significant differences depending on the existence and non-existence of SR_3_ and SR_4_ after six hours of contact with hydrochloric acid. In the non-existence of these inhibitors, iron & iron-oxide peaks are observed, whereas in their presence, oxide creation is delayed, and the intensity of the iron peak decreases. This illustrates the ability of SR_3_ and SR_4_ to prevent corrosion by encouraging the creation of a protective layer. The XRD method is crucial for analyzing the composition of corrosion products in steel before and after inhibitor treatment.

### 3.8. UV-visible

**Fig. 11:**
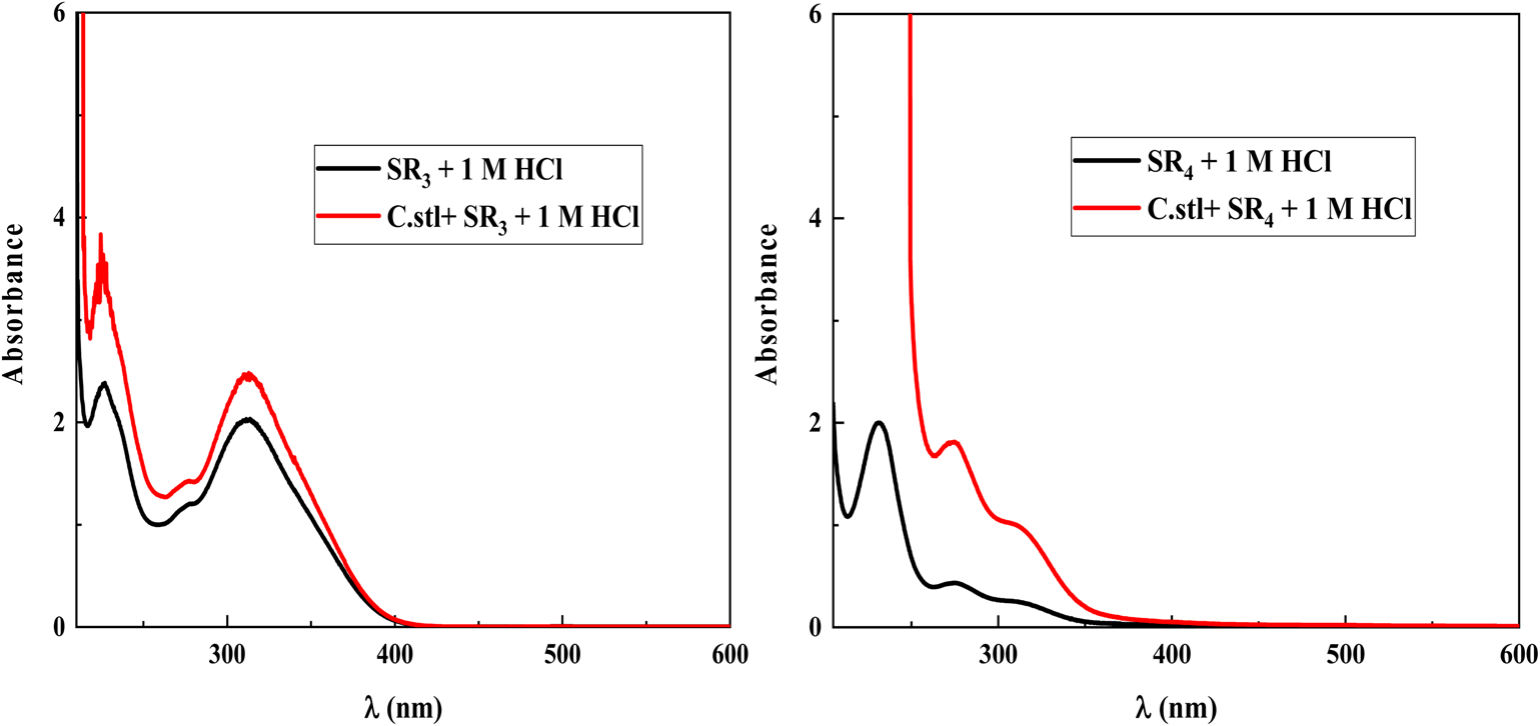
UV-visible spectra of SR_3_ and SR_4_ in the acid medium before (black) and after (red) 72 hours of C.stl quenching.

As seen in Fig. 11, UV-visible/absorption spectra were taken for a medium including 1 × 10^−3^ M of SR_3_ & SR_4,_ both before and after C.stl was soaked for 72 hours at 303 K to confirm the likelihood of complex creation between SR_3_, SR_4_, and Fe. According to earlier research, a wavelength shifts and/or a change in absorbance are signs of complex formation between C.stl and inhibitor molecules. The *n*–π* electronic transition caused by the ring is represented by a single band at around 231.1 nm for SR_4_ and two bands at about 225.7 nm and 312.5 nm for SR_3_ in the UV-visible spectra of SR_3_ and SR_4_ dissolved in an acidic medium before C.stl immersion. Following a 72 hours soak, these bands see a redshift and an increase in absorbance. These modifications imply that interactions between Fe^2+^ and benzodiazepine molecules have occurred in the acidic media.

### 3.9. Comparison of SR_3_ and SR_4_

According to the data summarized in Table 7, it is possible to confirm that SR_4_ has a better corrosion inhibition performance compared to SR_3_. SR_4_ has a lower corrosion current density, high inhibition efficiency, and polarization resistance, suggesting the formation of a more effective protective coating on the surface of carbon steel. Lower roughness and increased contact angle of SR_4_ are also observed through surface analysis, which indicates better surface protection. Moreover, the theory of more negative Δ*G*_ads_ of SR_4_ (−43.1 kJ mol^−1^) than that of SR_3_ (−40.7 kJ mol^−1^) indicates that it is adsorbed more on the steel surface. This could be because of the existence of the phenyl group in SR_4_, which increases the interaction of the molecule with the Fe surface.

**Table 7:** Comparative electrochemical and surface properties of SR_3_ and SR_4_ as corrosion inhibitors for carbon steel in 1 M

Parameter/method	SR_3_ (10^−3^ M)	SR_4_ (10^−3^ M)
Nomenclature	9-Ethyl-2,3,4,9-tetrahydrobenzo[*b*]cyclopenta[*e*][1,4]diazepin-10(1*H*)-one	1-Ethyl-4-phenyl-1*H*-benzo[*b*][1,4]diazepin-2(3*H*)-one
*i*_corr_ (µA cm^−2^)	133.3	63.5
*η*_PPD_ (%)	87.9	94.2
*R*_P_ (Ω cm^2^) (EIS)	144.4	300.7
*η*_EIS_ (%)	85.1	92.8
Surface roughness (nm)	70.7	21.5
Contact angle	99.8	102.1
Δ*G*_ads_	−40.7	−43.1

### 3.10. Theoretical analysis

#### 3.10.1. DFT calculations

**Fig. 12:**
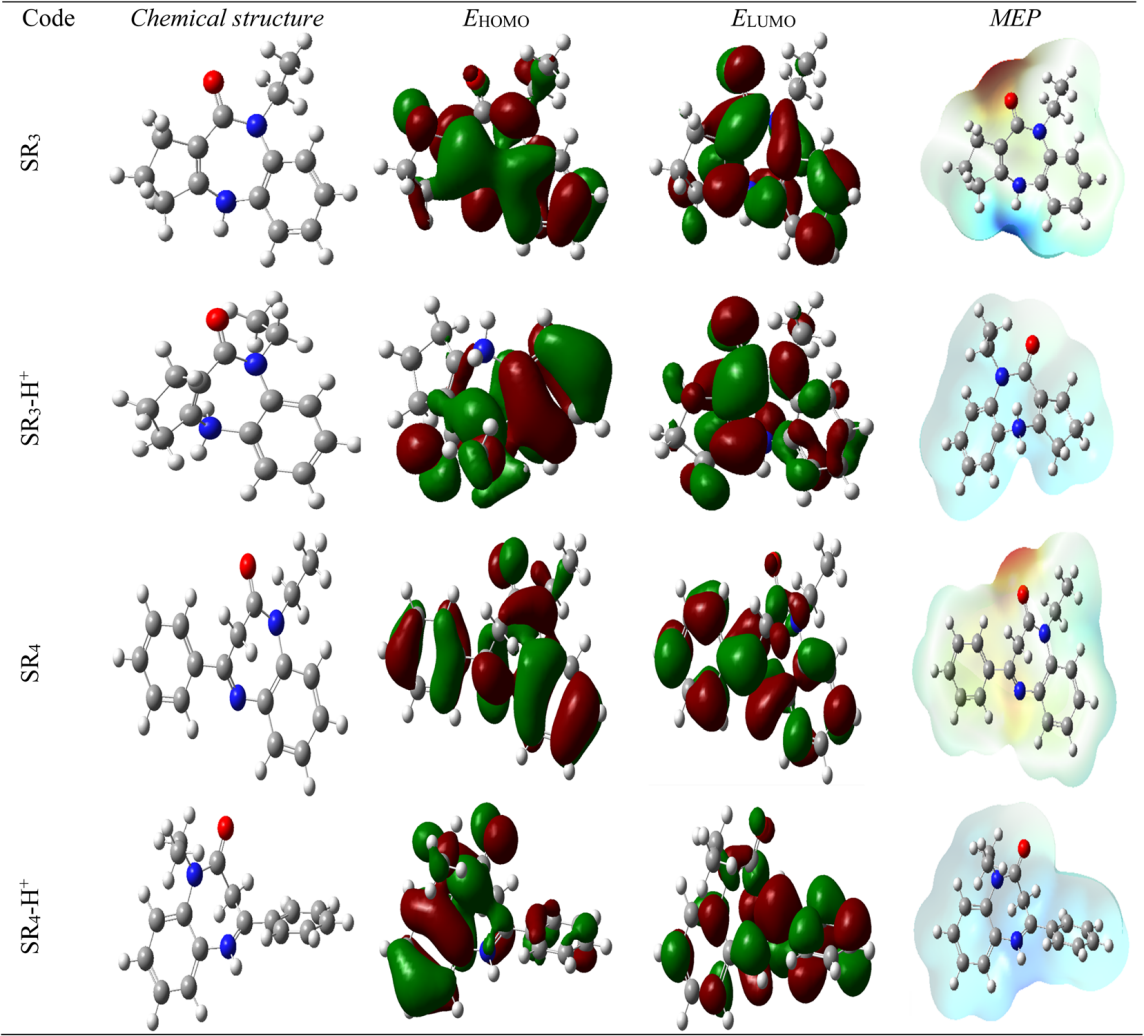
Molecular structure, (FMO), & (MEP) of the neutral and protonated forms of SR_3_ and SR_4_.

The DFT method's examination of an organic molecule's HOMO and LUMO orbitals' electron density distribution forms its foundation. Nowadays, many other global descriptors of chemical reactivity are calculated using this method.^40^ This electrical distribution can predict the compound's interaction with the metal/surface of interest.^41^ Corrosion-inhibiting compounds may interact with the adsorbing metal substrate to produce this reaction. These compounds' active sites are identified by their *E*_HOMO_ and *E*_LUMO_ values, which serve as electron donors to partially or filled orbitals on the metal surface.^42^ Inhibitor compounds with heteroatoms can bind to protons in acidic environments with a high proton (H^+^) content, changing the electron density distribution and impacting the mechanism of action. Fig. 12 shows (FMO), & (MEP) neutral and protonated forms of SR_3_ and SR_4_.

The reactivity of SR_3_ and SR_4_ molecules can be measured by measuring the energies of the border molecular orbitals (HOMO & LUMO). Table 8 displays the overall reactivity indices for SR_3_ & SR_4_'s neutral & protonated forms. It should be borne in mind that a molecule's propensity to give up electrons during interactions with metal orbitals increases with its *E*_HOMO_ value.^43–45^ An essential feature affecting the reactivity of a compound is its energy-gap (Δ*E* = *E*_LUMO_ – *E*_HOMO_). HOMO & LUMO interactions become stronger as this gap is reduced, increasing the reactivity of the compound, & conversely. According to our results, the neutral, protonated form of SR_4_ shows a reduced energy gap, indicating that it is more reactive than SR_3_. This could also mean that SR_4_ has a higher inhibitory efficiency. These observations offer convincing confirmation and are align with our experimental findings.

**Table 8:** Quantum-chemical/descriptors of SR_3_ & SR_4_ in their neutral & protonated forms

Descriptor	*E*_HOMO_ (eV)	*E*_LUMO_ (eV)	Δ*E* (eV)	*χ* (eV)	*η* (eV)	*σ* (eV^−1^)	Δ*N*_110_
SR_3_ neutral	−5.645	−1.145	4.500	3.395	2.250	0.444	0.316
SR_3_ protonated	−7.117	−1.953	5.164	4.535	2.582	0.387	0.055
SR_4_ neutral	−6.310	−2.016	4.293	4.163	2.147	0.465	0.153
SR_4_ protonated	−7.109	−3.343	3.765	5.226	1.883	0.531	−0.107

The values of Δ*N*_110_, 0.316 for SR_3_, 0.153 for SR_4_, 0.055 for SR_3_-H, and −0.1078 for SR_4_-H respectively, confirm this observation. Positive Δ*N*_110_ values suggest that neutral forms tend to give up electrons, while negative values indicate that charged compounds do not share electrons. *E*_LUMO_ values are also used to assess the attraction effect: a lesser value of this descriptor demonstrates a larger propensity of species to accept electrons. According to our findings, electrons are transferring from the HOMO of benzodiazepines to the metal/surface, as indicated by Δ*N*_110_ values below 3.6, which is consistent with the inferences made from the energy gap.^46^

Furthermore, other properties of the molecule being studied, such as absolute-electronegativity (*χ*) & hardness (*η*), influence the reactivity of this species. The interchange of electrons between the inhibitor compound & the metal/surface is also impacted by variations in electronegativity. Electrons tend to move from the protective compound, which is less electronegative, to the metal support, which is more electronegative, to balance chemical potentials.^47^ According to our findings, the protonated molecules SR_3_-H and SR_4_-H have larger *χ* values than the neutral forms, indicating that the charged/forms interact more with the treated surface (see Table 8). Additionally, the *η* parameters, which are also displayed in Table 8, indicate that the protonated/compounds are more reactive because they have lower values than their neutral counterparts.

In conclusion, quantum chemical calculations are proving very helpful. They significantly support the experimental findings and demonstrate that SR_4_ has a greater inhibitory capacity than SR_3_.

#### 3.10.2. Local reactivity (LR)

**Fig. 13:**
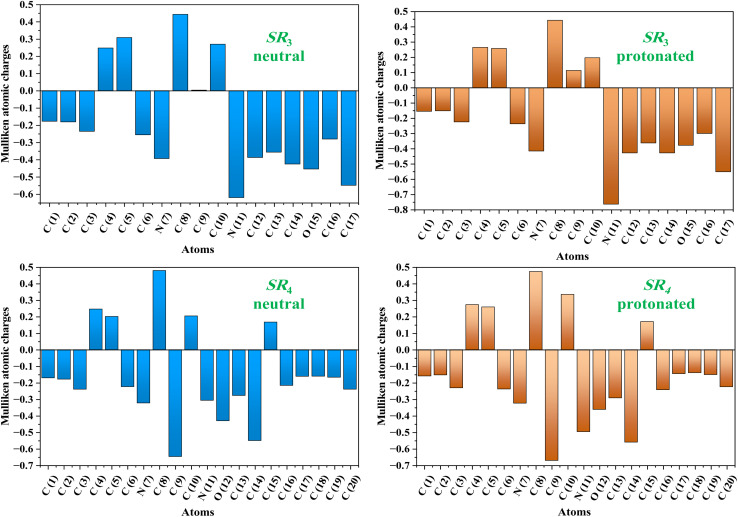
Mulliken atomic charges representations for the SR_3_ and SR_4_ molecules obtained by the DFT analysis in their neutral & protonated forms.

**Fig. 14:**
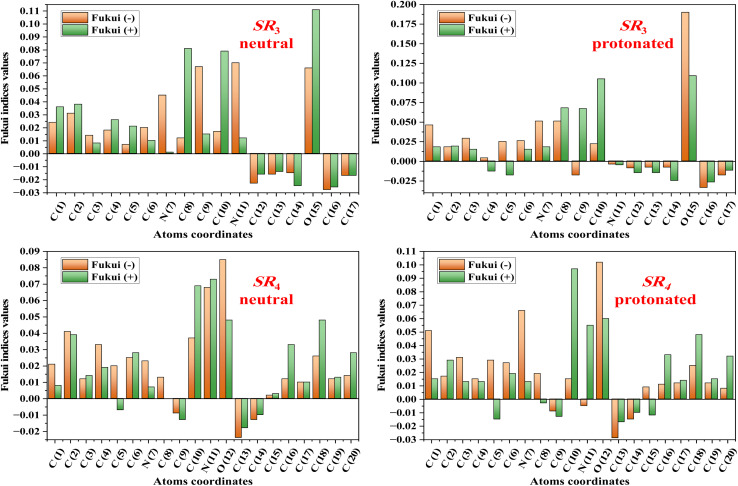
Fukui function representations for the SR_3_ and SR_4_ molecules obtained by the DFT analysis in their neutral and protonated forms.

To demonstrate improved numerical stability and electron distribution in compounds, the charge density of SR_3_ and SR_4_ was determined using the Mulliken charge atoms and Fukui indices.^48,49^ The Mulliken charge was chosen to understand the mechanisms of charge transfer in a compound through nucleophilic and electrophilic attack. The atoms' Mulliken charges are computed and shown in Fig. 13. All heteroatoms contain an abundance of electrons with negative charges, as can be seen from these results. The main purpose of this data is to determine how charges are positioned on the molecule's backbone.^50^ Through a donor–acceptor process, the negatively charged heteroatoms are adsorbed to the metallic substrate, forming a coordinating bond. In their interactions with the metallic surface, these atoms function as nucleophilic sites. It has been noted that oxygen, nitrogen, and carbon atoms in aromatic rings have an excess negative charge. This characteristic lowers the pace of corrosion by enabling the particles to be adsorbed/C.stl/surface. Highlighting the reactive sites of each organic molecule resulting from the Fukui functions is one of the best aspects of the LR section.^51,52^ This allows one to determine the electrophilic and nucleophilic locations from the Fukui descriptors for the neutral & protonated forms of SR_3_ & SR_4_ molecules, which are used in this study to prevent the corrosion of the C.stl/surface in an acidic environment of hydrochloric acid. These indices are displayed in Fig. 14. Every atom with a significant Fukui (+) value functions as an electrophilic location, and every atom with a high Fukui (−) value functions as a nucleophilic site, according to the literature. The atoms with significant numbers in the neutral form of the SR_3_ molecule are C(8), C(10), & O(15); in the protonated form of the SR_3_ molecule, they are C(8), C(9), C(10), & O(15); and in the neutral and protonated form of the SR_4_ molecule, they are C(10), N(11), O(12), C(16), & C(18).^53^ These findings are shown in Fig. 14. High Fukui (+) values indicate that a site is most vulnerable to nucleophilic attacks, while high Fukui (−) parameters indicate that a site is most prone to electrophilic attacks.^54^ Ultimately, these findings demonstrate that the investigated inhibitor molecules are more reactive, enabling them to effectively prevent C.stl from corroding in the corrosive atmosphere containing 1 M HCl.^55,56^

#### 3.10.3. MD simulation

**Fig. 15:**
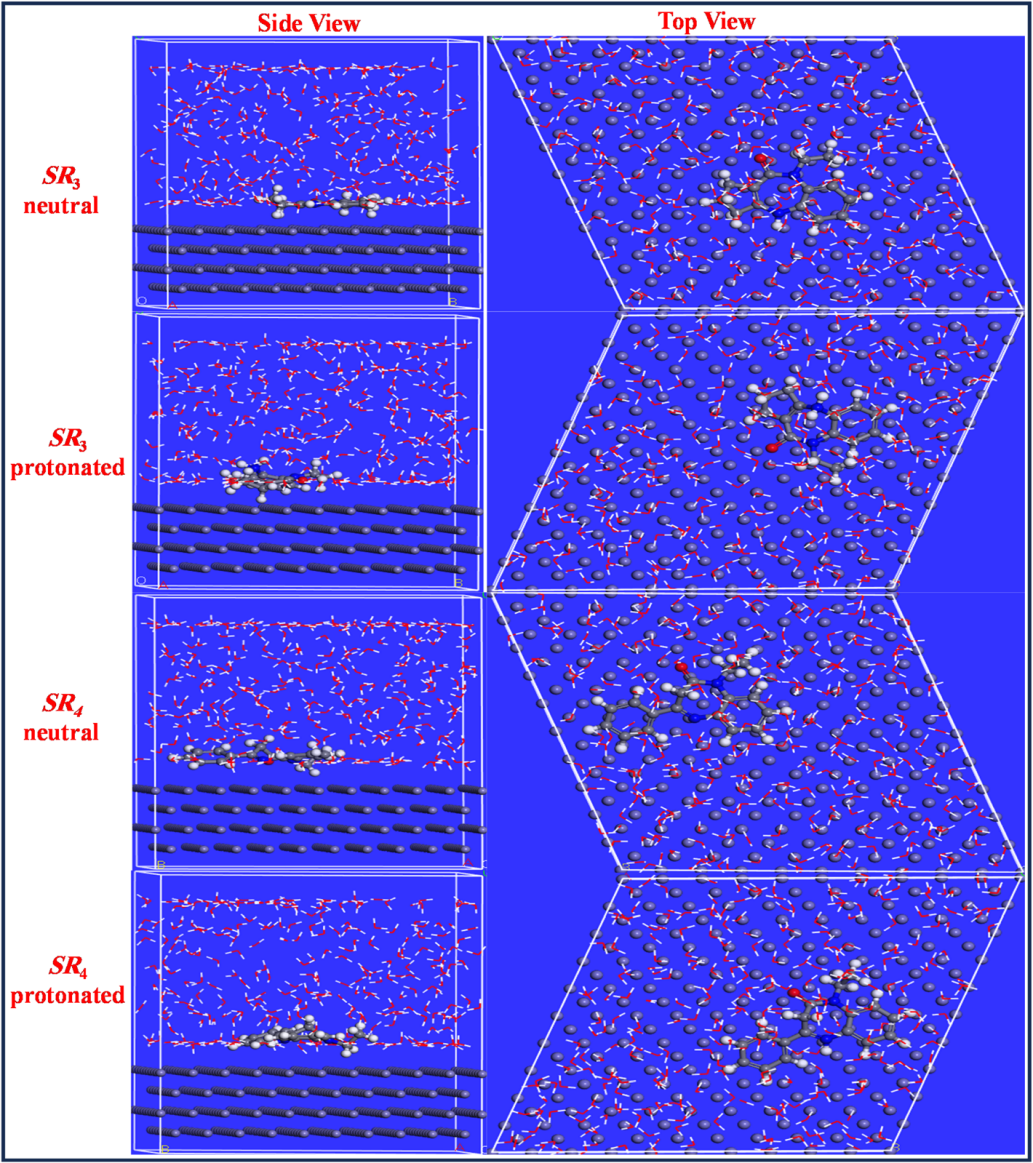
Top & side views of the SR_3_ & SR_4_ adsorbed on the Fe(110) surface in neutral and protonated solutions.

The interactions between the neutral and protonated forms of the SR_3_ and SR_4_ inhibitor particles and the Fe(110) surface have been further investigated using molecular dynamics simulation (MDS), as illustrated in Fig. 15. The neutral and protonated versions of the SR_3_ and SR_4_ inhibitors adsorb almost to the Fe(110)/surface, where π-electrons in aromatic rings and heteroatoms can make chemical interactions (Fig. 15).^57,58^ The following equations can be used to determine the interaction and binding energies between the tested inhibitors and the Fe(110) surface once the adsorption process has reached equilibrium:^59,60^

7 *E*_interaction_ = *E*_total_ − (*E*_surface+solution_) + *E*_solution_

8 *E*_binding_ = −*E*_interation_

where the system's entire energy is represented by *E*_total_. *E*_inhibitor+solution_ is the total energy of the inhibitor and solution, while *E*_surface+solution_ is the total energy of the Fe(110) surface and solution without the inhibitor. The total energy of the solution is called *E*_solution_. Fig. 15 displays the top and side views of the adsorption configuration of each evaluated compound as determined by MD simulations. The associated interaction energy (*E*_interation_) values for neutral SR_3_, neutral SR_4_, protonated SR_3_, and protonated SR_4_ are −512.7, −533.1, −451.4, and −477.6 kcal mol^−1^, respectively. Throughout the molecular dynamic modeling process, the inhibitor compounds gradually transferred closer to the Fe(110)/surface until, as seen in Fig. 15, they assumed a completely horizontal, flat orientation.^61,62^ This orientation allows the inhibiting molecules to attain high inhibitory efficiency by covering a greater area of the metal surface.^63,64^

### 3.11. Adsorption mechanism

The results of the study indicate that organic compounds act as corrosion inhibitors by attaching themselves to the metal surface. The adsorption of molecules onto the metal can occur through various mechanisms. Retrodonation involves the interaction of the molecule's π electrons with the metal surface, while chemisorption results from the interaction between the non-bonding electron doublets of the molecules and the metal. Physisorption, on the other hand, is governed by electrostatic interactions between charged species present in the medium.

In an acidic environment, nitrogen-containing compounds can exist in neutral or protonated form. Furthermore, oxidation of the metal surface leads to a loss of electrons, giving it a positive charge. This situation then promotes the adsorption of negative ions, such as chloride ions, onto the metal surface.^65^

**Fig. 16:**
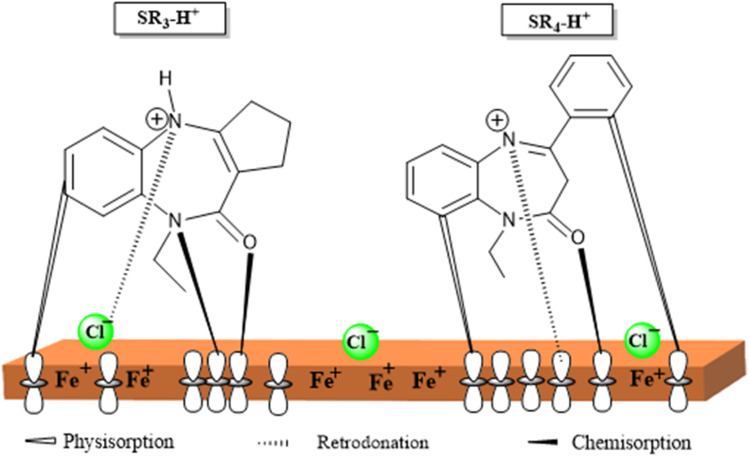
Schematic illustration of the adsorption mechanism of SR_3_ and SR_4_ on the C.stl surface in 1 M HCl.

The adsorption mechanism of SR_3_ and SR_4_ on the carbon steel surface in 1 M HCl is illustrated in Fig. 16. In an acidic medium, the inhibitor molecules become protonated, forming SR_3_H^+^ and SR_4_H^+^ species. Chloride ions (Cl^−^) in the solution are first adsorbed onto the positively charged steel surface, thereby creating negatively charged sites. The adsorption of SR_3_H^+^ and SR_4_H^+^ occurs through a mixed mechanism involving both physisorption and chemisorption. Physisorption results from electrostatic interactions between the protonated inhibitor molecules and the negatively charged surface. Chemisorption occurs through donor–acceptor interactions between the lone pair electrons of nitrogen and oxygen atoms, as well as the π-electrons of aromatic rings, and the vacant d-orbitals of Fe atoms. In addition, retrodonation from the metal surface to the inhibitor molecules may further strengthen the adsorption and stabilize the protective film formed on the steel surface.

## 4. Conclusion

The effectiveness and inhibitory mechanisms of benzodiazepine derivatives in halting the corrosion of C.stl in a hydrochloric Acid environment are assessed in this study using a variety of methodologies. The compounds' exceptional efficacy as inhibitors was demonstrated by their highest performance of 87.9% for SR_3_ and 94.2% for SR_4_ at the ideal concentration. The Langmuir isotherm governs the chemical adsorption of SR_3_ & SR_4_/C.stl, where they function as a mixed inhibitor. While UV-visible analysis of the environment demonstrated the production of complexes, surface investigation using SEM-EDS, AFM, contact angle, and XDR, verified the development of a protective layer on the steel/surface. Molecular-dynamics (MD) simulations display that SR_3_ & SR_4_ adsorb in parallel to the Fe(110) iron surface, boosting corrosion-protection, whereas the findings of DFT predictions correlate significantly with experimental performance.

However, several limitations should be considered. The inhibition efficiency of SR_4_ decreases with increasing temperature, suggesting limited thermal stability under elevated temperatures. SR_3_ provided incomplete protection, as residual corrosion was observed in SEM analyses. Additionally, all experiments were conducted over short-term immersion periods (up to 72 hours), and long-term stability under industrially relevant conditions has not been assessed. These limitations highlight the need for further studies to fully validate the practical applicability of these inhibitors.

## Conflicts of interest

There are no conflicts to declare.

## Data Availability

All supporting data are contained within the manuscript.
